# Opening the box of PANTHORA in Alzheimer’s disease

**DOI:** 10.1038/s41392-022-01204-7

**Published:** 2022-10-02

**Authors:** Martin Korte, Reinhard W. Köster

**Affiliations:** 1 Technische Universität Braunschweig, Zoological Institute, Div. Cellular Neurobiology, Spielmannstr. 7, 38106 Braunschweig, Germany; 2 Helmholtz Centre for Infection Research, AG NIND, Inhoffenstraße 7, 38124 Braunschweig, Germany; 3 Technische Universität Braunschweig, Zoological Institute. Div. Cellular and Molecular Neurobiology, Spielmannstr. 7, 38106 Braunschweig, Germany

**Keywords:** Diseases of the nervous system, Cellular neuroscience

A study published in Nature Neuroscience indicates that autophagy dysfunction in neurons precedes the formation of amyloid plaques.^1^ These findings ask for a reconsideration of the conventionally accepted sequence of events in plaque formation in Alzheimer’s disease.

Alzheimer’s Disease (AD) with synaptic dysfunction and cognitive decline is a devastating age-associated neurodegenerative disease accounting for about two thirds of all cases of dementia.^2^ The affected brain structures are histologically marked by intracellular neurofibrillary tangles (NFTs) and extracellular amyloid plaques of deposited β-amyloid peptides (Aβ), derived by proteolytic processing of the extracellular domain of the amyloid precursor protein, APP.^2^ The plaques appear to form first, while NFTs are indicative for an advanced state of AD, but their interrelation is still under dispute.^3,4^ Therefore, it is extensively investigated why, how, where and when, Aβ plaques are first formed in AD-affected brains that are associated with astro-/microgliosis. These neuroinflammatory processes serve to protect or remove degenerative cells and attempt to clean out amyloid plaques. Can AD-affected neurons contribute with a malfunctioning protein degradation mechanism to this process? A recent elegant study by Lee and coworkers^5^ has analyzed in several genetic mouse models of AD the major cytoplasmic recycling process of cells termed autophagy.

**Fig. 1:**
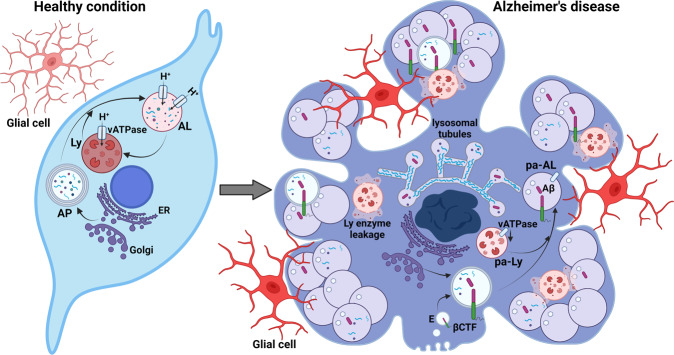
PANTHOS Progression. This graphic depicts how poorly acidified autolysosomes (AL) (purple) congregate in the soma of neurons (left), pushing out the cell membrane to form petal like structures, so called PANTHOS (right). Lysosomes (Ly), the cells disassembly organelle, begin leaking proteases (pink) and Aβ fibrils (gray squiggles) aggregate in lysosomal tubules around the cell nucleus. By this means a packed neuron collides with nearby PANTHOS cells and ruptures (right), recruiting glia cells to turn the Aβ debris into larger plaques. pa-AL: poorly acidified AL; poorly acidified Lys (pa-Ly). Adapted after Lee et al., Nature Neuroscience, 2022^1^

In this self-eating process, double-walled membranes enclose intracellular components in forming vesicles, termed autophagosomes,^5^ which subsequently fuse with endosomes and eventually lysosomes (autolysosomes) for content degradation and its release for recycling (Fig. 1).

Multiplex imaging revealed that AD-affected neurons - already long before symptomatic stages and plaque formation - display defects in autophagy, in which autolysosomes form, but fail to properly acidify due to compromised vATPase activity.

These deacidified autolysosomes with impaired degrading lysosomal enzyme activity accumulate in the cytoplasm, start to fuse to larger autophagic vacuoles and eventually incorporate further endomembrane system structures such as Golgi and ER, probably because of an excessive demand for membrane material. These AD neuron-specific vacuoles extend from the perinuclear region into petal-like structures termed PANTHOS (greek: poisonous flower) and expand the cellular membranes to blebs. Such PANTHOS-containing neurons were identified by the authors in post-mortem human AD brain samples as well.

APP, the source of A*β*-peptide deposition, is transported to the membrane through the secretory pathway along the endomembrane system,^1^ thus ER incorporation into PANTHOS structures provides these autophagic vacuoles with significant amounts of APP that can be proteolytically processed in endosomes/lysosomes. Strikingly, Lee et al. demonstrate that PANTHOS structures contain amyloid plaques with increasing levels of maturation over time concomitant with a compromised neuronal morphology.

Early PANTHOS-containing neurons are not associated with reactive astrocytes or activated microglia. Therefore, initial autophagic defects, deacidified autolysosome accumulation, PANTHOS build-up and initial plaque formation evades detection by inflammation-mediating neighboring cells. Only when PANTHOS-containing neurons lose structural integrity, they are surrounded by activated astrocytes/microglia. At this advanced stage PANTHOS-containing neurons merge to larger structures likely giving rise to extracellular senile plaques that are eventually released into the extracellular space of the brain.

These findings of the authors imply several ground-breaking new insights into the possible mechanism of AD-associated plaque formation. Foremost, amyloid plaque formation considered to occur extracellularly due to APP proteolysis turns into an intracellular process followed by a release of already formed and matured plaques into the extracellular environment.^4^ Consequently, plaque formation should be viewed primarily as a cell-autonomous rather than a non-cell autonomous process.

The generation and accumulation of deacidified autolysosomes occurs long before histological signs of plaque formation and at stages when neurons appear to be healthy at large. This cellular metabolic signature could probably be exploited for diagnostic purposes of initiating AD or related neurodegenerative diseases at stages when preventive measure would still have major effects.

Based on the author’s finding the view on neuroinflammatory mechanisms in association with AD may be seen from a different perspective. The first contribution of reactive glia to AD progression may be a late one, when amyloid plaques have already formed and matured inside PANTHOS-containing neurons. But by promoting neuronal cell death of late-stage PANTHOS-neurons, glia might serve as a can opener causing the release of plaque material into the brain environment. This would promote plaque spread, turns further plaque growth from an intracellular into an extracellular process and enables plaque contact and influence possibly with glia.

Besides providing crucial novel insights into cell biological processes of amyloid plaque formation, like any exciting research new important question arise. What is so far missing, is a careful assessment about the role of neuroinflammation, since the authors did not elucidate precisely when neuroinflammation starts and what it contributes to the observed phenotype. It also remains unclear which mechanism mediates reduction in vATPase-activity, and why some autophagosomes are poorly acidified, while others show a properly acidic pH-milieu. Autophagy is a process common to all cells. Why are cholinergic neurons of the forebrain affected first by AD, are they more sensitive to PANTHOS formation? Reestablishing proper acidic conditions in poorly acidified autolysosomes in AD mouse models at early stages should prevent PANTHOS-formation and subsequent establishment of histological features as well as AD symptoms in these animals. Such a rescue would further provide evidence that amyloid plaque formation is initiated in a cell-autonomous process by autophagy defects in neurons themselves. This could trigger a plethora of new drugs or even the development of preventive strategies.

Neurofibrillary tangles as a hallmark of late AD stages do not appear in PANTHOS-neurons until stages of their destruction. Are these deficits inherent to the genetic mouse models that are unable to phenocopy NFT formation like in human AD brains? To investigate the relationship between PANTHOS-forming processes and neurofibrillary tangle appearance will be an interesting area of research.

Overall the findings described in Lee et al.^1^ strongly support the notion that, months before plaques develop, Aβ accumulates in faulty lysosomes neurons, and that this finally disturbs neuronal functioning and might cause neuronal death, leaving behind amyloid plaques. This supports therefore the hypothesis that lysosome dysfunction is an early, causal, and, most importantly, pathogenic process in AD.
